# Clinical and laboratory evidence of Haff disease – case series from an outbreak in Salvador, Brazil, December 2016 to April 2017

**DOI:** 10.2807/1560-7917.ES.2017.22.24.30552

**Published:** 2017-06-15

**Authors:** Antonio C Bandeira, Gubio S Campos, Guilherme S Ribeiro, Cristiane W Cardoso, Claudilson JC Bastos, Tiago L Pessoa, Karine A Araujo, Maria Fernanda R Grassi, Alessandra P Castro, Rejane H Carvalho, Ana Paula P B Prates, Luana L Gois, Veronica FD Rocha, Silvia I Sardi

**Affiliations:** 1 Faculdade de Tecnologia e Ciencias – FTC - Medical School, Salvador, Brazil; 2 Hospital Aliança, Salvador, Brazil; 3 Instituto Gonçalo Moniz, FIOCRUZ, Salvador, Brazil; 4 Virology Laboratory, Federal University of Bahia, Salvador, Brazil; 5 Instituto de Saúde Coletiva, Federal University of Bahia, Salvador, Brazil; 6 Secretaria Municipal de Saúde, Salvador, Brazil; 7 Hospital Aeroporto, Salvador, Brazil; 8 Universidade Catolica do Salvador, Salvador, Brazil; 9 Hospital Geral Roberto Santos, Salvador, Brazil

**Keywords:** Haff disease, outbreak, acute rhabdomyolysis, Brazil

## Abstract

We describe a series of 15 Haff disease cases from an outbreak in Salvador, Brazil, starting early December 2016. Eleven cases were grouped in four family clusters of two to four individuals, four were isolated cases. All but one patient consumed cooked fish; 11 within 24h before symptoms onset. Cases consumed ‘Olho de Boi’ (*Seriola* spp.) and ’Badejo’ *(Mycteroperca* spp.). A total of 67 cases were detected, the last case was reported on 5 April 2017.

We describe a case series of Haff disease causing an outbreak in the city of Salvador, Brazil. The first case was reported on 1 December 2016 and here we present the clinical and epidemiological data of the first 15 cases of the 67 total cases detected as at 5 April 2017. On 14 December, after the identification of the first six cases, the local public health authorities began an active search for new cases in the health units and hospitals in Salvador and also searched medical records to retrospectively identify cases compatible with Haff disease back to July 2016. Structured interviews were conducted with every affected patient since the beginning of the outbreak.

## Case definition

We defined cases as patients presenting with (i) sudden onset of muscle pain in more than two body regions e.g. superior limbs, inferior limbs, neck/trapezium region, back region, thorax and abdominal region, not related to intense physical activity; (ii) elevated levels of creatinine phosphokinase (CPK) (more than fivefold above the upper limit of the normal reference value of 170 U/L (i.e. 850 U/L) within 24 hours of presentation; and (iii) patients who recalled an ingestion of fish or fish products within 72 hours before the onset of symptoms.

## Laboratory investigations

Serial measurements of muscle enzymes, such as creatine phosphokinase (CPK), aspartate aminotransferase (AST), alanine aminotransferase (ALT) and lactate dehydrogenase (LDH) were performed in all patients. In addition, troponin I levels and creatine kinase-MB (CK-MB) fractions were measured in three and two cases, respectively.

We screened 10 patients for chikungunya and Zika virus RNA (serum n=4, urine n= 3, saliva n=3) given the significant circulation of both viruses in Salvador since 2015 [1,2]. RNA was extracted using QIAmp Viral RNA mini kit, according to manufacturer’s instructions. Reverse transcription (RT)-PCRs were performed using standard protocols [3,4]. RT-PCR analyses were also performed for enteroviruses and Parechoviruses using reagents from AccessQuick (Promega), according to standard protocols [5-8]. Thirteen samples (serum and faeces) from eight patients were tested. Sequencing was performed by a sequencing facility using the ABI-Prism 3500 Genetic Analyzer (Applied Biosystems) on 40 ng of products and 2 pmol of each primer. The results were analysed with the help of the Basic Local Alignment Search Tool (BLAST).

Pieces of uncooked fish (‘Olho-de-Boi’) from one patient were sent for toxin analysis at the Food and Drug Administration (FDA), in the United States (US), through the Brazilian Ministry of Health.

## Outbreak description

The outbreak started on 1 December 2016 with a family cluster of four patients who presented to the emergency department (ED) within less than 24 hours with sudden onset of muscle pain in different body parts, initiated at the trapezium area, and followed by dark urine in two. They all had consumed the same fish products 12 to 72 hours before symptoms onset and all had highly elevated muscle enzymes, compatible with rhabdomyolysis. Eight days later, two other non-linked family clusters of three and two individuals, respectively, came within hours to the ED with a similar history of ingestion of fish and symptoms of rhabdomyolysis. Within each cluster, all patients had consumed the same fish. During the following three weeks, three more cases (comprising another family cluster of 2 cases) presented the same symptoms, had the same history of fish ingestion, summing up to 12 cases by 28 December 2016. In January 2017, three further cases were diagnosed, adding up to the 15 cases studied in detail and presented here.

The majority of the 15 cases of Haff disease reported here clustered between the epidemiological week 48 and 51 of 2016, however, new cases continued to be reported up to epidemiological week 14 of 2017 (Figure). After the recognition of the outbreak in December 2016, local health authorities started an active search for cases and they identified similar cases admitted to local hospitals in Salvador back in July 2016.

**Figure:**
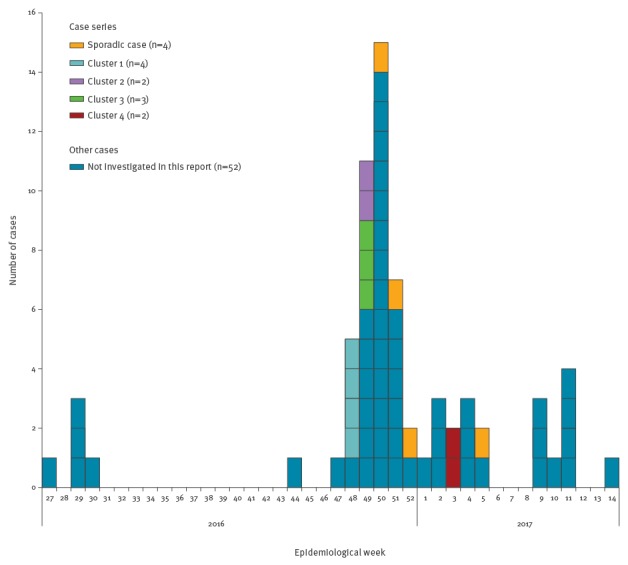
Case series of reported cases of Haff disease according to the epidemiological week of symptoms onset, Salvador, Brazil, 1 December 2016–31 January 2017 (n=15)

The figure shows Haff disease cases identified retrospectively and prospectively after recognition of the outbreak.

The median age of the first 15 cases was 43 years (range: 14-75), there were slightly more men (n=9) than women (n=6), and all cases reported sudden onset of muscle pain, particularly, pain in the neck or trapezium regions was reported by 14 patients. Eight patients reported dark urine compatible with myoglobinuria and a mild rash was seen in four. At initial medical care or during hospitalisation, none of the cases presented with fever, arthralgia, respiratory, or gastrointestinal symptoms, except for two patients who reported transient loose stools. Six patients had comorbidities. Thirteen patients were hospitalised with a median hospital stay of 3 days. Clinical and demographic variables are shown in Table 1.

**Table 1:** Demographic and clinical characteristics of cases in outbreak of Haff disease, Salvador, Brazil, 1 December 2016–31 January 2017 (n=15)

Characteristics	Number or median (range)
Male	9
Age, years	43 (14–75)
Symptoms/signs at first medical care^a^	
Neck/Trapezium pain	14
Arms pain	14
Thighs or legs pain	13
Back pain	10
Thoracic pain	2
Abdominal pain	2
Throat pain	1
Muscle weakness	6
Fever	0
Rash	4
Dark urine	8
Vital signs	
SBP, mmHg^b^	146 (110–168)
DBP, mmHg^b^	79 (60–89)
Heart rate, bpm^b^	74 (60–97)
Comorbidities^a^	
Arterial Hypertension	1
Hyperlipidaemia	2
Type II diabetes mellitus	1
Chronic atrial fibrillation	1
Depression	2
Hypothyroidism	1
Patients on statin therapy	2
Outcome	
Hospitalisation	13
Acute kidney injury	2
Death	0

Bpm: beats per minute; DBP: diastolic blood pressure; SBP:  systolic blood pressure.

^a^ Patients may present with more than one comorbidity or symptom/signs.

^b^ Data available for 11 patients.

CPK levels at presentation ranged from 743 to 105,755 U/L and decreased rapidly on subsequent days in parallel with the improvement of symptoms. Troponin I levels were less than 0.034 ng/mL in all patients with measurements. CK-MB fraction ranged from 1.9% to 6.3%.

RT-PCR for chikungunya and Zika viruses, as well as nested RT-PCR for Parechovirus, yielded negative results for all patients in all types of samples. The RT-PCR for enterovirus generated non-specific products in four samples. However, upon analysis of the sequenced fragments no virus could be identified.

### Epidemiological links, food consumption history and symptom onset

In the interviews, patients were asked about possible links to other cases, fish consumption and the timely relation to symptom onset. Eleven cases were grouped in four family clusters of two to four individuals and four were isolated cases. One limitation of the present study is that we did not investigate all family members to determine the attack rates within clusters. Table 2 shows the clustering pattern for the cases, as well as the species of fish consumed by each of them, and the time elapsed between fish ingestion and symptom onset. All but one case had consumed cooked fish. Time between consumption and onset of symptoms ranged between 2 to 5 and 72 hours. Of the 14 cases who reported fish consumption, 11 had ingested fish within 24h before symptoms onset.

**Table 2:** Case clustering, species of fish consumed, and time elapsed between fish ingestion and initial symptoms onset for case series of Haff disease patients, Salvador, Brazil, 1 December 2016–31 January 2017 (n=15)

Case number	Cluster number	Type of fish consumed	Time elapsed between fish ingestion and symptoms onset
Case 1	Cluster 1	A + B	48 – 72 hours
Case 2	Cluster 1	A + B	48 – 72 hours
Case 3	Cluster 1	A + B	12 – 24 hours
Case 4	Cluster 1	A + B	48 – 72 hours
Case 5	Cluster 2	B	21 hours
Case 6	Cluster 2	B	11 hours
Case 7	Cluster 2	B	7 hours
Case 8	Cluster 3	A	12 hours
Case 9	Cluster 3	A	13 hours
Case 10	No cluster	B	10 hours
Case 11	Cluster 4	Unknown^a^	7 hours
Case 12	Cluster 4	Unknown^a^	6 hours
Case 13	No cluster	A	10 hours
Case 14	No cluster	None^b^	NA
Case 15	No cluster	A	2 – 5 hours

A: ‘Olho de Boi’ (*Seriola* spp.); B: ‘Badejo’ (*Mycteroperca* spp); NA: not available.

^a^ Unknown type of fish consumed.

^b^ Case 14 reportedly did not consume fish, but ate local Afro-Brazilian food, which might have fish by-products used in its preparation.

The species of seawater fish consumed were ‘Olho de Boi’ (*Seriola* spp) and ‘Badejo’ (*Mycteroperca* spp). One case had reportedly not eaten fish, but eaten a local Afro-Brazilian dish with possible fish by-products used in its preparation within 24 hours of symptoms onset.

Analysis of the fish sample by the FDA revealed no toxins or heavy metal contamination.

## Background

Cases of Haff disease were first described in 1924, in the Baltic region of Prussia and Sweden and involved the consumption of different cooked freshwater fish, such as burbot (*Lota lota*), pike (*Esox* sp.) and freshwater eel (*Anguilla anguilla*) [9]. In the US, the first cases of Haff disease were reported in Texas in 1984, and hereafter the Centers for Disease Control and Prevention (CDC) reported six additional cases of Haff disease in patients from California and Missouri, who had eaten buffalo fish [10]. In 2001, two cases were identified in North Carolina who had eaten baked Atlantic salmon [11] and in 2014, further individual case reports were published, both cases developed symptoms after the ingestion of buffalo fish [12,13]. In 2010, China reported cases associated with the ingestion of crayfish [14].

In Brazil, an outbreak of 27 cases of Haff disease occurred during 4 months in the northern state of Amazonas in 2008 [15] and an additional case from the Amazon region was reported in 2013 [16].

It is considered that the condition is due to an unknown toxin in the aquatic food chain.

## Discussion

We describe a cases series in an outbreak of Haff disease in Salvador that involved 15 patients as at 31 January 2017 with the majority of them clustered in families. An earlier outbreak of Haff disease in our country was reported in the Amazon region in 2009. It was related to ingestion of three different species of freshwater fish present in the Amazonian rivers [15] and authors identified the same pattern of family clusters as observed by us [15].

An important limitation of case series is the methodological limitation concerning risk factor analysis and the control of possible confounders. However, the sudden onset of similar symptoms in family clusters, a few hours after consumption of the same food, point to a common exposure. The absence of fever and gastrointestinal symptoms and laboratory signs of infection (data not shown) led us to hypothesise an ingested toxin as the cause. Given the universal ingestion of fish or its by-products by all cases and the compatibility with the described Haff syndrome, we considered a fish toxin as the most probable cause of illness in our case series.

After its recognition, seven cases of Haff disease were retrospectively identified back to July 2016, and 60 cases have been reported since 1 December 2016, totalling 67 cases reported in this outbreak until now. The last case was reported on 5 April 2017 [17].

‘Olho de Boi’ (*Seriola* spp.) and ‘Badejo’ (*Mycteroperca* spp.) are seawater fish. Palytoxin is a toxic substance present in some soft corals (*Palythoa* sp.) and analogues were also isolated from dinoflagellates of the genus *Ostreopsis* that are vastly distributed in tropical water throughout the world, including the coast of Brazil [18]. Our patients did not present with typical manifestations of palytoxin intoxication i.e. chest pain, shortness of breath, wheezing, tachycardia, although two patients reported episodes of loose stools (diarrhoea may occur in palytoxin intoxication) and all had myalgia and rhabdomyolysis, a common feature of palytoxin intoxication related to the Na+-K+-ATPase binding effect. Samples of fish consumed by one of our patients were analysed for ciguatoxin by the FDA with negative results. This is not surprising in the face of the difficulty in isolating the aetiological toxin involved. In 21 similar cases in the US, where samples of suspected fish or seafood were tested by the CDC or the FDA, all were negative for different aquatic toxins, including ciguatoxin, saxitoxin, brevetoxin, tetrodotoxin, palytoxin, and cyanobacterial toxins [19].

It is important for travel medicine physicians to be alert in case of patients returning from Salvador, Brazil, with myalgia and symptoms of rhabdomyolysis to consider Haff disease as a possible differential diagnosis. Care should also be taken when treating these patients so as to avoid non-steroidal anti-inflammatory agents because of possible concurrent renal toxicity [20].
